# CSF Surfactant Protein Changes in Preterm Infants After Intraventricular Hemorrhage

**DOI:** 10.3389/fped.2020.572851

**Published:** 2020-09-25

**Authors:** Matthias Krause, Wolfgang Härtig, Cynthia Vanessa Mahr, Cindy Richter, Julia Schob, Joana Puchta, Karl-Titus Hoffmann, Ulf Nestler, Ulrich Thome, Matthias Knüpfer, Corinna Gebauer, Stefan Schob

**Affiliations:** ^1^Department of Neurosurgery, University Hospital Leipzig, Leipzig, Germany; ^2^Paul Flechsig Institute for Brain Research, Medical Faculty of University Leipzig, Leipzig, Germany; ^3^Department of Neuroradiology, University Hospital Leipzig, Leipzig, Germany; ^4^Department of Ophthalmology, University Hospital Leipzig, Leipzig, Germany; ^5^Department of Neonatology, University Hospital Leipzig, Leipzig, Germany

**Keywords:** intraventriclar hemorrhage, hydrocephalus, surfactant, preterm, cerebrospinal fluid

## Abstract

**Introduction:** Surfactant proteins (SP) have been shown to be inherent proteins of the human CNS and are altered during acute and chronic disturbances of CSF circulation. Aim of the study was to examine the changes of surfactant protein concentrations in CSF of preterm babies suffering from intraventricular hemorrhage.

**Patients and Methods:** Consecutive CSF samples of 21 preterm infants with intraventricular hemorrhages (IVH) and posthemorrhagic hydrocephalus (PHHC) were collected at primary intervention, after 5–10 days and at time of shunt insertion ~50 days after hemorrhage. Samples were analyzed for surfactant proteins A, B, C, and G by ELISA assays and the results were compared to 35 hydrocephalus patients (HC) without hemorrhage and 6 newborn control patients.

**Results and Discussion:** Premature patients with IVH showed a significant elevation of surfactant proteins SP-A, C, and G compared to HC and control groups: mean values for the respective groups were SP-A 4.19 vs. 1.08 vs. 0.38 ng/ml. Mean SP-C 3.63 vs. 1.47 vs. 0.48 ng/ml. Mean SP-G 3.86 vs. 0.17 vs. 0.2 ng/ml. SP-A and G concentrations were slowly falling over time without reaching normal values. SP-C levels declined faster following neurosurgical interventions and reached levels comparable to those of hydrocephalus patients without hemorrhage.

**Conclusion:** Intraventricular hemorrhages of premature infants cause posthemorrhagic CSF flow disturbance and are associated with highly significant elevations of surfactant proteins A, C, and G independent of total CSF protein concentrations.

## Introduction

Surfactant proteins (SPs) have been demonstrated to be inherent proteins of the CNS (1–3) and are expressed with different distribution patterns of SP-A and SP-D compared to SP-B and SP-C. The more rheologically active SP-B and SP-C were observed in choroid plexus and ependymal cells of the brain and spinal canal, representing the major sites of CSF formation and the CSF-tissue interface. In parallel the opsonins SP-A and SP-D are found at the sites of the blood-brain and the blood-CSF barrier, respectively (3). Furthermore, the SPs are also present in significant concentrations in cerebrospinal fluid (CSF) (2, 4, 5).

Prematurely born babies have a high risk of intraventricular hemorrhage (IVH), which is presumed to arise from immature germinal matrix at the ventricular walls of the lateral ventricles. Our recent studies focusing on embryology and concerning the CNS surfactant system in hydrocephalic conditions suggest a relationship between surfactant protein concentrations and intraventricular hemorrhage (6). Immature lung suffers from higher surface tension under postpartum breathing. An immature brain may also be subjected to undue pulsatile forces caused by insufficient CNS surfactant concentrations at the CSF-brain interface. In newborn rats, SP-B and SP-D showed a marked delay in occurrence of immunoreactivities at the brain-CSF and blood-brain-interfaces compared to lung tissue of the same animal (1). Both surfactant proteins appear only in adult rat brains, whereas SP-A and SP-C can already be found in embryologic stages. Thus, rheologically active SP-B may play a role in an incompetent reduction of surface tension within the pulsating brain: Resulting higher shear forces may lead to rupture of vulnerable vascular structures within the ependymal zones of the germinal matrix, thus leading to hemorrhage in similarity to premature lung pathology (7). Because the influence of breathing on cerebral pulsation seems to exceed the arterial pulsation, postpartum adaptation may explain the higher incidence of IVH within the 1st days after birth compared to the incidence of prenatal hemorrhages.

SP-A has recently found to modulate CNS immune response of astrocytes and microglial cells (6–8). Additionally, we have demonstrated that SP-G plays a role in CNS water hemostasis and is elevated in inflammatory and hydrocephalic conditions (9).

The aim of this study was the examination of potential alterations of surfactant protein concentrations within the CSF of neonates suffering from intraventricular hemorrhage resulting in posthemorrhagic hydrocephalus (PHHC).

## Patients and Methods

### Patients

We investigated serial CSF specimens of 21 neonatal patients suffering from hydrocephalus due to intraventricular hemorrhage. All patients or caregivers gave their written informed consent for the scientific use of CSF samples and analysis of clinical and radiological data.

All neonatal patients required neurosurgical intervention to relieve intracranial pressure due to posthemorrhagic hydrocephalus (PHHC) either by implanting an external ventricular drainage (*n* = 11), a ventricular access device for repeated tapping to remove CSF (*n* = 1) or an endoscopic lavage procedure plus external ventricular drainage (*n* = 9). When applicable, follow-up CSF was examined after 5–10 days in patients with external ventricular drainage (EVD) or Rickham-Reservoir (PHHC 5–10 days, *n* = 13) and at time for shunt insertion at a median of 57 days after birth (PHHC shunt, *n* = 7). Four additional patients were treated in other institutions initially but required transfer to our center for shunt insertion due to persisting CSF disturbance. Thus, for those four patients only CSF specimen at time of shunt insertion were available.

Indication for primary intervention due to PHHC was made either due to signs of elevated intracranial pressure (apneas, bradycardia) or rapid increase of head circumference. At the coronal ultrasound plane all patients presented a ventricular size of at least one lateral ventricle larger than 2 cm^2^ as minimum area (correlating to ~5–6 mm above the 97th percentile of Levine's ventricular index). Early interventions were performed esp. in patients that underwent neuroendoscopic lavage procedures to alleviate the posthemorrhagic inflammatory response and resolve obstructions of the aqueduct or third ventricle. Mean gestational age was 28.5 weeks (range 23.7–41 weeks) and birth weight 1,356 g (range 560–3,970 g). At the time of primary intervention, the mean age of the patients was 22 days. All patients suffered from Levine IVH grades II or more and developed ventriculomegaly.

The control group comprised six neonatal patients that necessitated CSF examination by lumbar puncture to rule out CNS infection. Mean gestational age was 32.3 weeks (range 31–41 weeks) and birth weight 2,326 g (range 1,370–3,780 g). Furthermore, CSF samples were obtained from 35 older patients (mean age 13.5 years ranging from 0 to 65.9 years) that underwent a diagnostic workup and treatment of hydrocephalus in isolated aqueductal stenosis without hemorrhagic or postinfectious cause (HC group) (1). An overview of all demographic data is given in Table 1.

**Table 1:** Overview of demographic data of the patient cohorts.

	**Control**	**PHHC initial**	**PHHC 5–10 days**	**PHHC shunt**	**HC**
*N*	6	21	13	7	35
Mean age at time of intervention (Range)	8 days (2–15 days)	22 days (18–25 days)	29 days (26–33 days)	57.4 days (48–66 days)	13.5 yrs (1 day−65.9 yrs)
Sex (m/f)	4/2	12/9	7/6	4/3	16/19
SP-A CSF level (ng/ml)	0.38 (0.14–0.62)	4.19 (3.68–4.71)	3.87 (3.14–4.60)	2.92 (2.12–3.73)	1.08 (0.79–1.36)
SP-B CSF level (ng/ml)	0.27 (0–0.69)	6.2 (5.15–7.24)	3.91 (2.56–5.26)	5.27 (3.61–6.92)	0.14 (0–0.67)
SP-C CSF level (ng/ml)	0.48 (0–0.94)	3.63 (2.84–4.41)	1.95 (0.91–2.99)	1.77 (0.73–2.81)	1.47 (0.88–2.06)
SP-G CSF level (ng/ml)	0.20 (0–0.44)	3.86 (3.42–4.3)	1.96 (1.38–2.54)	1.12 (0.56–1.68)	0.17 (0–0.51)
Total CSF protein (mg/ml)	0.46 (0.34–0.85)	2.20 (1.12–3.18)	1.95 (1.59–2.31)	1.4 (1.2–1.61)	0.11 (0.09–0.13)
CSF Cell Count	8 (2–16)	774 (7–1,759)	36 (0–395)	22 (0–218)	9 (4–17)
CSF Red Blood Cell Count	1 (0–7)	310 (7–950)	16 (0–45)	5 (0–22)	2 (0–4)

The neonatal groups are separated according to the age of the babies at the time of intervention (postnatal days).

*PHHC, posthemorrhagic hydrocephalus; PHHC initial, CSF obtained at first intervention; PHHC 5–10 days, CSF obtained during follow-up routine examination; PHHC shunt, CSF obtained at time of shunt insertion ~50 days after hemorrhage. HC, hydrocephalus. yrs, years. All concentrations presented are mean values with confidence interval in brackets*.

### Methods

Quantification of surfactant protein concentrations was performed using enzyme-linked immunosorbent assays (ELISA) according to the manufacturer's manual. Commercially available ELISA kits (USCN, Wuhan, China) were used to quantify the amount of SP-A (E90890Hu, ELISA Kit for Surfactant Associated Protein A), SP-B (E91622Hu, ELISA Kit for Surfactant Associated Protein B), SP-C (E91623Hu, ELISA Kit for Surfactant Associated Protein C) and SP-G (E90890Hu, ELISA Kit for Surfactant Associated Protein G) in CSF samples. The analyses were performed using a microplate spectrophotometer (ELISA-reader) at a wavelength of 450 nm and a reference wavelength of 405 nm for measuring the absorbance. Surfactant protein concentrations in ng/ml CSF were calculated by comparison between standard series and the determined values of antigen concentration (protein concentration) according to the manufacturer's manual.

Routine CSF laboratory data (cell counts, CSF lactate and glucose concentrations, total CSF protein, and protein electrophoresis as well as bacterial cultures) were obtained to rule out infection or other inflammatory and autoimmune diseases. Patients with CSF infections were excluded from further analysis. Total CSF protein content and SP concentrations were analyzed with Pearson's correlation coefficient to rule out a direct association between total protein content and surfactant proteins.

Statistical analyses were performed with the SPSS Version 22 (SPSS Inc., Chicago, IL, USA) based on the Welch *t*-test and ANOVA. Thereby, the significance level was set to 0.01.

This study was approved by the institutional review board of the medical faculty (Ethikkommission Universität Leipzig Az 330-13-18112013).

## Results

Analyses of the CSF obtained in 21 primary interventions in newborns revealed marked elevations of CSF surfactant proteins A, C, and G in PHHC patients compared to control and HC group (all *p* < 0.001). There was no association between total CSF protein content and SP-concentrations with R values 0.14 for SP-A, 0.08 for SP-C, and 0.0009 for SP G, respectively (**Figure 3**).

SP-B was detected in only 1 of 6 (16.7%) controls and 7 of 35 (20%) HC group specimens and only 3 (14%) PHHC patients. Therefore, SP-B results are shown in the table but not incorporated in the boxplot figures.

**Figure 1:**
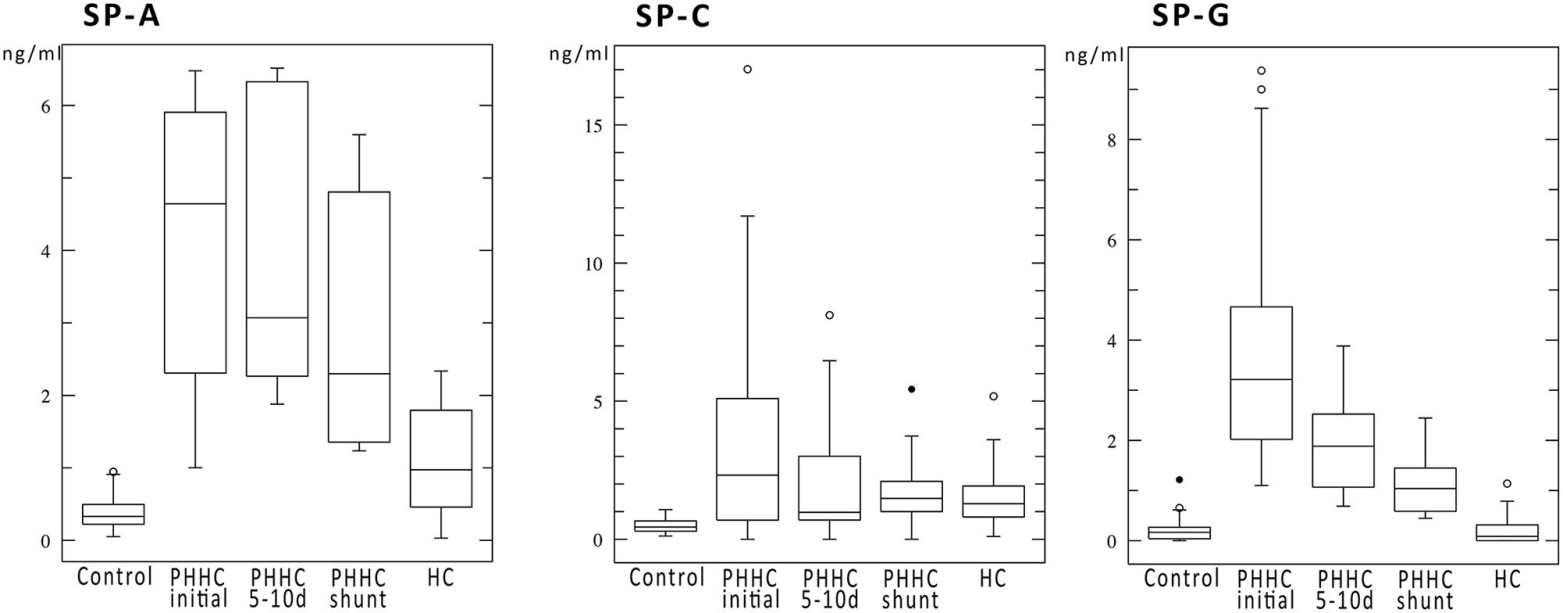
Boxplots show the surfactant protein concentrations in ng/ml of SP-A, SP-C, and SP-G. All SPs showed a marked elevation after IVH compared to control and HC. SP-C levels decreased over time to values comparable to hydrocephalus patients. SP-G declined but remained elevated at time of shunt insertion.

**Figure 2:**
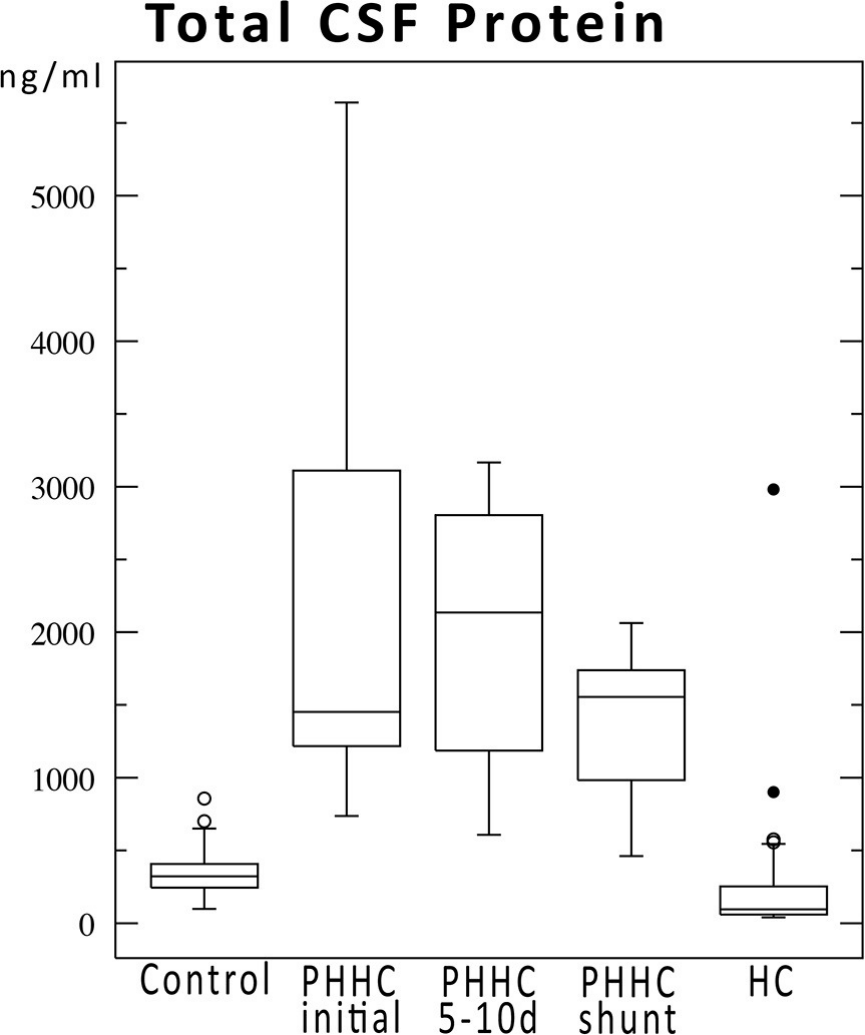
Total CSF protein concentrations over time for IVH patients compared to control and hydrocephalus cohorts. Protein concentrations declined after hemorrhage but did not reach normal values until shunt implantation ~50 days after hemorrhage.

During follow-up, SP concentrations fell over time, but did not reach control levels. SP-C fell to HC group levels after the primary intervention, whereas all other SPs still remained markedly elevated compared to HC group levels at time of shunt surgery approx. 50 days after hemorrhage (Figure 1). Total concentration of CSF protein was very high after hemorrhage at time of first intervention and declined over time without reaching normal values at time of shunt implantation (Figure 2). SP-A appeared to be massively elevated in all analyzed preterm babies at all time points after IVH and at time of shunt insertion.

## Discussion

To the best of our knowledge the present study demonstrates for the first time that surfactant protein concentrations in the CSF of neonates suffering from intraventricular hemorrhage are elevated. Compared to non-hemorrhagic hydrocephalus caused, e.g., by aqueductal stenosis, surfactant proteins A, C, and G are significantly elevated. The few preterm and newborn patients (*n* = 6) making up the control group had normal concentrations of SPs compared to pediatric and adult control individuals (2). Thus we do not consider the SP elevation to be a result of preterm delivery.

Currently, there is only limited knowledge of the surfactant system in the developing CNS of rats (6). SP-A and C have been found to be first to be expressed in embryonic rat brains whereas SP-B and SP-D were detected in adult rat brains only. Due to the paucity of data, it remains hypothetical that immaturity of the cerebral surfactant system—in analogy to the pulmonary—plays a pathophysiological role in the development of preterm IVH. Further research needs to elucidate those questions.

Asymptomatic control group SP concentrations in preterm and newborn babies are unknown because examinations require lumbar puncture. Due to that ethical aspect, the small control group is one limitation of the study. However, the time course of SP concentrations is independent from control group comparison. In a previously published study, a significant negative age correlation of surfactant protein concentrations was only found for SP-A over a time span from birth to 80 years (1).

Surfactant protein elevation might be a result of blood contamination by the hemorrhagic components. However, our previous studies demonstrated by mRNA analysis and immunohistochemistry a clear CNS borne production of surfactant proteins on ependymal surfaces and within the cerebral parenchyma at the perivascular spaces (2, 6). There was also no association between the concentrations of total CSF protein content and SPs in the current cohort, indicating that SP levels are not dependent from the amount of CSF blood contamination alone. This is in concordance with other previously published results in children and adults (1).

Furthermore, CNS borne expression of all SPs has been shown by immunohistochemical data (1, 2, 9, 10). A disturbed or unbalanced expression of SPs might play a causative role in IVH development, and the observed elevations during acute phase situations then mirror the initial disbalance overlaid by secondary effects. The theory of incompetent surfactant layer function at the origin of undue periventricular shear stress as mechanism of IVH development is supported by our findings in rat brain development: SP-B and SP-D were detected in adult rats only, but not in embryonic stages (1, 7, 11).

SP-G has been proposed to be an acute phase-associated and inflammation-mediating CNS surface-active protein (9, 12). In the present study, its concentration declines over time but does not reach normal values of neither controls nor hydrocephalus patients. This supports a role of SP-G in removal of debris and inflammatory responses in the time course of the patients, as postulated in our previous study (9).

Notably, there is an undeniable SP-A and SP-G difference between posthemorrhagic hydrocephalus patients and patients with hydrocephalus due to other reasons (Figure 1). Similar to SP-G concentrations, SP-A levels are strongly elevated with a reduction during the follow-up period. Both SP-G and SP-A may play a role in modulating rheological properties of CSF and paravascular clearance of toxic metabolites after hemorrhage via the glymphatic pathway (13–15). As demonstrated by Diler et al. (16) and Mittal et al. (12) SP-G, SP-A, and the novel SP-H promote phagocytosis of blood degradation products and adjust viscosity and surface tension by interaction with CSF lipids (11, 12, 16, 17).

SP-C levels were reduced within the follow-up period to levels of hydrocephalus patients without hemorrhagic origin. As postulated previously, SP-C may be elevated downstream due to mechanical stress and consecutive increase of intracranial pressure (3–5). Of note, two patients with low SP-C concentrations at 5–10 days after primary intervention did not require shunt surgery. It remains unclear, whether surfactant proteins might offer any prognostic potential to predict persistent CSF circulation disturbances in posthemorrhagic hydrocephalus. However, a prospective investigation with higher numbers of patients and a defined sampling protocol is needed to confirm this hypothesis.

**Figure 3:**
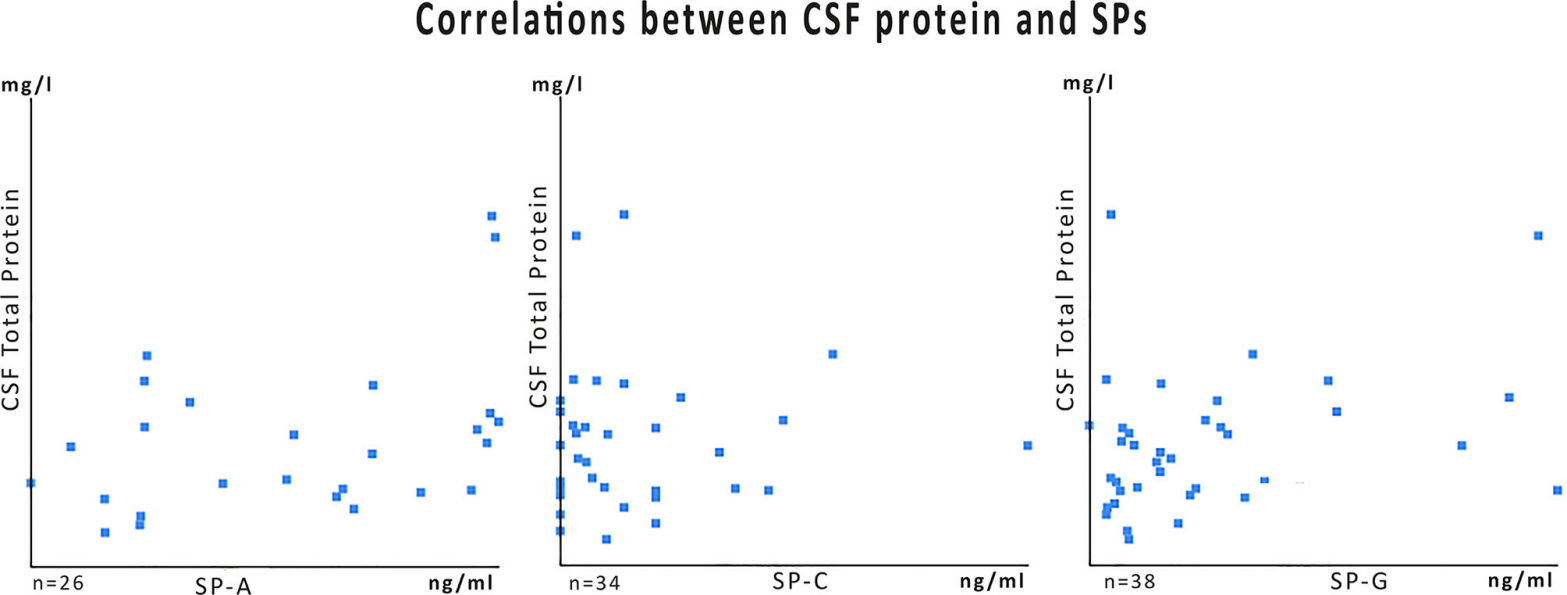
Data plots for SP-values of the PHHC group in relation to the total CSF protein content. The numbers of specimens (*n*) differ because samples from initial treatment, follow-up, and shunt surgery were cumulated. For some samples total protein content was not available from clinical routine data charts. Correlation coefficients (*R*) were very low: 0.14 for SP-A, 0.08 for SP-C, and 0.0009 SP-G.

Surfactant protein concentrations change over time independently from total CSF protein but depending on their putative functions (Figure 1 vs. Figure 2). A reduction of CSF proteins after hemorrhage is the natural course of the condition. Treatment of CSF circulation disturbance will lead to elution of excess CSF protein and cells, normalize intracranial pressure, and alleviate ventricular volume load. The primary CSF concentrations prior to intervention underline the CNS-borne production of SPs and make a secondary effect due to blood contamination unlikely, as previously shown in other studies (1, 6, 9) (Figure 3).

The present study is limited by various shortcomings. This work is based on a retrospective collection of data without a stringent sampling protocol, because sampling was only possible during clinically indicated procedures. Thus, the global parameters (ICP, blood pressure, oxygenation, systemic inflammation) at time of CSF sampling are unknown. Investigations focused on these parameters require and justify the use of animal models. Secondly, the control group and hydrocephalus group consists of older patients that SP-D was not examined due to small sample volumes that did not allow further ELISA measurements. SP-B was found in only few controls and hydrocephalus patients but was detected in very high concentrations in preterm babies after IVH. The few specimens, however, did not allow further detailed analysis, and future investigations are necessary to evaluate SP-B.

A major shortcoming is the lack of surface tension analysis and lipid studies of the corresponding CSF specimen to underline the clinical significance of surfactant in hydrocephalic conditions. Reduction of surface tension by protein concentration and cellular components have been stated already in the 1940s in historical books and were demonstrated by Kratochvil and Hrncír (18) in 2002. However, this requires further clinical evaluation and investigation. Additonal investigations in adult IVH or subarachnoid hemorrhage are warranted to compare adult and neonatal pathophysiology.

In light of the multiplicity of evidence of the existence of the Cerebral Surfactant System and the importance of surfactant application in the respiratory distress syndrome of the preterm, further research may open horizons to diagnostic and therapeutic use of CSF surfactant proteins in preterm infants.

## Conclusion

Intraventricular hemorrhages of the preterm and newborn babies that cause posthemorrhagic CSF disturbance are associated with highly significant elevation of surfactant proteins A, C, and G, independent from total CSF protein concentrations.

SP-A and G concentrations fell slowly over time without reaching normal values. In contrast, the reduction of SP-C levels following neurosurgical interventions occurred faster. SP-C reached levels similar to those of hydrocephalus patients that did not suffer from ventricular hemorrhage. This suggests an involvement of SPs in the acute and subacute posthemorrhagic CNS response. Further investigations are needed to elucidate potential diagnostic and therapeutic approaches to treat this specific pathology.

## Data Availability Statement

The raw data supporting the conclusions of this article will be made available by the authors, without undue reservation.

## Ethics Statement

The studies involving human participants were reviewed and approved by Ethikkommission Universität Leipzig Az 330-13-18112013. Written informed consent to participate in this study was provided by the participants' legal guardian/next of kin. The present study included the retrospective analysis of clinical data, routine CSF examinations, and surfactant protein analysis. It was positively reviewed by the local ethics committee of University Leipzig (Ethikkommission Universität Leipzig Az 330-13-18112013).

## Author Contributions

All authors reviewed the data and critically revised the manuscript. MKr, UN, UT, MKn, and CG collected CSF samples, clinical data, and routine CSF data. UN and MKr prepared all CSF samples for further analysis. JP and SS performed the ELISA analysis. MKr and CM conducted statistical analysis.

## Conflict of Interest

The authors declare that the research was conducted in the absence of any commercial or financial relationships that could be construed as a potential conflict of interest.
